# Dorsal root ganglia CX3CR1 expressing monocytes/macrophages contribute to arthritis pain

**DOI:** 10.1016/j.bbi.2022.09.008

**Published:** 2022-11

**Authors:** Silvia Oggero, Chiara Cecconello, Rita Silva, Lynda Zeboudj, George Sideris-Lampretsas, Mauro Perretti, Marzia Malcangio

**Affiliations:** a Wolfson Centre for Age Related Diseases, King’s College London, London SE1 1UL, UK; b The William Harvey Research Institute, Barts and The London School of Medicine, Queen Mary University of London, London EC1M 6BQ, UK; c Centre for Inflammation and Therapeutic Innovation, Queen Mary University of London, London, UK

**Keywords:** Macrophages, Monocytes, Arthritis, Fractalkine, CGRP, Pain

## Abstract

Pain is a persistent symptom of Rheumatoid Arthritis, and the K/BxN serum transfer model recapitulates both association and dissociation between pain and joint inflammation in RA. Furthermore, this model features monocyte/macrophage infiltration in joints and lumbar dorsal root ganglia (DRG), where these immune cells are close to nociceptive neurons. We focussed on CX_3_CR_1_-monocyte/macrophage trafficking and show that at peak paw swelling associated with nociception, CX_3_CR_1_ deletion altered neither swelling nor macrophage infiltration/phenotype in paws. However, acute nociception and DRG non-classical monocyte numbers were reduced in CX_3_CR_1_^GFP/GFP^ (KO) compared to CX_3_CR_1_^+/GFP^ (WT). Nociception that persisted despite swelling had resolved was attenuated in KO and correlated with DRG macrophages displaying M2-like phenotype. Still in the DRG, neurons up-regulated neuropeptide CGRP and olcegepant treatment reduced acute swelling, nociception, and leukocyte infiltration in paws and DRG. We delineate *in-vitro* a signalling pathway showing that CGRP liberates the CX_3_CR_1_ ligand fractalkine (FKN) from endothelium, and in bone marrow-derived macrophages, FKN promotes activation of intracellular kinases, polarisation towards M1-like phenotype and release of pro-nociceptive IL-6. These data implicate non-classical CX_3_CR_1_-expressing monocyte and macrophage recruitment into the DRG in initiation and maintenance of arthritis pain.

## Highlights

• Lack of CX_3_CR_1_ impairs DRG monocyte infiltration and modulates macrophage phenotype.
• The peptide CGRP in DRG sensory neuron increases at peak inflammatory allodynia.
• Olcegepant prevents leukocytes recruitment in paws and DRG and allodynia.
• CGRP liberates CX_3_CR_1_ ligand fractalkine from HUVEC.
• Fractalkine releases pronociceptive IL-6 from primary macrophages.

## 1 Introduction

Pain is a persistent feature of rheumatoid arthritis (RA). It is associated with the flares of RA and can persist even when joint inflammation is controlled by current anti-arthritic drugs. To advance our understanding of the mechanisms underlying RA pain and identify novel analgesic strategies, we employ models of RA that mimic both association and dissociation of pain with inflammation as they constitute an ideal platform to investigate mechanisms of acute and persistent nociception. Specifically, in the K/BxN model of inflammatory arthritis, a few days after passive immunization, mice exhibit hind paw nociceptive hypersensitivity (allodynia) in concomitance to clinical scores (paw swelling) (Christianson et al., 2010). However, allodynia persists after swelling has spontaneously resolved at around 4 weeks from immunization (Allen et al., 2020, Christianson et al., 2010, Woller et al., 2019). In this model, joint pathology is driven by neutrophils and macrophages and the infiltration of non-classical monocytes into the joints is crucial for the initiation, progression, and resolution of inflammation (Misharin et al., 2014). Monocytes are recruited to the synovium during the early phase of the model, where they display plasticity and differentiate into macrophages *in situ*, with changes from macrophage M1-like (pro-inflammatory) to M2-like (anti-inflammatory) phenotype over time. Notably, resident synovial macrophages possess a protective M2-like phenotype throughout the time course of the model (Culemann et al., 2019), highlighting the importance of recruited cells for initiation and progression of arthritis. We have recently observed that monocytes/macrophages also populate the dorsal root ganglia (DRG) and can be found in vicinity of the cell bodies of sensory neurons innervating the joint (Allen et al., 2020). Within DRG, macrophage interaction with sensory neurons contributes significantly to peripheral mechanisms of nociception via release of pro-inflammatory mediators such as cytokines and prostaglandins (Allen et al., 2020, Massier et al., 2015, Segond von Banchet et al., 2009). Here we investigated the modality governing monocyte/macrophage invasion and modulation of nociceptive signalling in the DRG under inflammatory arthritis conditions.

Since monocytes/macrophages express CX_3_CR_1_ receptors, which mediate monocyte adhesion and transmigration via binding to endothelial chemokine CX_3_CL_1_ (Fractalkine, FKN) (Geissmann et al., 2003, Montague et al., 2018, Old et al., 2014), we hypothesised that the FKN/CX_3_CR_1_ pair plays a functional role in mediating macrophage communication with nociceptive neurons in DRG under inflammatory arthritis conditions. Although CX_3_CR_1_ is expressed by dendritic cells which play functional roles in *trans*-endothelial mechanisms (Vollmann et al., 2021), dendritic cells are not critical for K/BxN arthritis progression (Wu et al., 2007) and were not examined in this study. Instead we examined neutrophils which do not express CX_3_CR_1_ (Jung et al., 2000), but play a critical role in K/BxN paw inflammation (Norling et al., 2016).

## 2 Methods

### 2.1 Animals

8–12-week-old male and female CX_3_CR_1_^+/GFP^ (used as WT) and CX_3_CR_1_^GFP/GFP^ (used as KO) littermate mice (B6.129P2(Cg)-Cx3cr1^tm1Litt^/J) were used for experiments according to the United Kingdom Animals (Scientific Procedures) Act 1986 and following the guidelines of the Committee for Research and Ethical Issues of the International Association for the Study of Pain. All experimental study groups were randomized and blinded. Mice were housed in groups of up to 5 per standard cage. All animals were kept at room temperature with a 12-h light/dark cycle. Animals received food and water ad libitum.

### 2.2 Induction of K/BxN serum transfer inflammatory arthritis

Induction of K/BxN serum-transfer model of inflammatory arthritis was induced as previously described (Allen et al., 2020, Norling et al., 2016). Mice were injected with 50 μl of arthritogenic serum on days 0 and 2. Control mice received equivalent volume injections containing pooled sera from KRN/C57 mice (non-arthritic). Clinical signs of arthritis were evaluated using a 12-point scoring system. Each limb was scored separately; 0 to 3 points per limb with the following criteria: 0, no sign of redness/swelling; 1, redness/swelling observed in either ankle/wrist, pad, or any of the digits; 2, redness/swelling in 2 regions; and 3, redness/swelling seen in all limb sections. Scores for all 4 limbs were combined to give a maximum score of 12 points per animal. Ankle thickness was measured using a digital caliper. Ankle joint measurements were acquired before the first injection of serum and repeated daily after injection.

### 2.3 Drug

Olcegepant (Tocris Biosciences, Abingdon, UK) was dissolved in 2.5 % dimethyl sulfoxide in saline and injected at 1 mg/kg intraperitoneally, daily for 6 days, starting one day prior injection of K/BxN serum. Mechanical thresholds were assessed using von Frey filaments at 3 h after each injection.

### 2.4 Behavioural tests

Hind paw mechanical thresholds were assessed by application of calibrated von Frey monofilaments (0.02–1.0 g) to the hind paw plantar surface. Testing started with the application of a 0.07 g filament and each paw was assessed alternately between application of increasing stimulus intensity until a withdrawal response was achieved or application of 1.0 g filament failed to induce a response, to avoid tissue damage. The 50 % paw withdrawal threshold (PWT) was determined by increasing or decreasing stimulus intensity and evaluated using Dixon’s “up–down” method. Experiments were performed blind.

### 2.5 Flow cytometry of paw tissue

Leukocytes were isolated from arthritic and control paws after tissue digestion. Paws were cut 3 mm above the heel, skin from the feet was removed, and fingers disarticulated by pulling with blunt forceps. The foot was then incubated in 15 ml digestion buffer (Collagenase D [Roche; 0.5 μg/ml] and DNAse [Sigma-Aldrich; 40 μg/ml, Gillingham, UK] in serum-free RPMI) with gentle agitation for 30 min at 37 °C. Cells released during digestion were filtered through 70 μm cell strainer and kept on ice adding 2 ml of ice cold FBS (FBS qualified HI, Gibco). An additional 15 ml of digestion buffer was added to the foot and incubated for further 30 min at 37 °C. Liberated cells were passed through a 70 µm cell-strainer and combined with previous cells. Combined cells were centrifuged for 10 min at 400 xg and resuspended in phosphate buffer solution (PBS, Sigma) for staining. Cells were first incubated with anti-CD16/CD32 (eBioscience, 1:1000, 15 min, 4) antibody to block unspecific Fc receptor binding and then stained with Fixable Viability Dye eFluor™ 780 (Thermofisher, 1:1000) to identify live cells, while distinct leukocyte subtypes were identified using the following antibodies: CD45 BV450 (0.5 μg/ml, clone 30-F11, BD Biosciences), CD11b PE-Cy7(0.2 ug/mL, clone M1/70, ThermoFisher scientific), CD115 APC (2 µg/ml, clone AFS98, Biolegend), Ly6C (1 µg/ml, clone HK1.4, ThermoFisher scientific), Ly6G PE (0.5 μg/ml, clone 1A8, BD Bioscience), CD43 BV510 (1 mg/ml, clone S7, BD Bioscience), MHCII AF700 (1.25 μg/ml, clone M5/114, Biolegend), F4/80 BV650 (2 μg/ml,clone BM8, ThermoFisher scientific), CCR_2_ BV711 (0.5 µg/ml, clone 475301, BD Bioscience), CD206 BV605 (3 μg/ml, clone C068C2, Biolegend). Samples were run using LSRFortessa cell analyser (BD Bioscience) and analysed using FlowJo software (v10.1; Tree Start, Inc). All gating strategies were generated using Fluorescent Minus One (FMO) controls (Supplementary Fig. 1).

### 2.6 Flow cytometry of dorsal root ganglia

Flow cytometry for dorsal root ganglia tissue was performed at day 5 and 25 after K/BxN injections. Animals were *trans*-cardially perfused with saline solution and from each mouse, L3-5 DRG were dissected bilaterally, in accordance with the sensory innervation of rodent hind paws. DRG tissue were then dissociated using 3mg/ml dispase (Roche), 0.1 % collagenase (Sigma-Aldrich) and 200U/ml DNAse I (Roche) in F-12 medium (Life Technologies) for 45 min. DRG were triturated and then centrifuged for 5 min at 300 *xg*. Cell pellets were then resuspended in 100 µl PBS^-/-^ (no calcium chloride or magnesium chloride, Sigma). Cell suspensions were first incubated with Zombie NIR™ Fixable Viability kit (BioLegend) for 15 min at room temperature, and then incubated on ice for 20 min with anti-mouse CD16/CD32 (Clone 2.4G2, BD Biosciences, San Jose, CA) to block Fc receptors in PBS^-/-^ plus 2 % Bovine Serum Albumin (BSA) and 2 mM EDTA. Cells were washed and centrifuged for 1 min at 300 *xg* and then incubated with a mix of the following fluorochrome-conjugated anti-mouse antibodies for 30 min on ice: CD45.1-BV786 (5 µg/ml, clone 30-F1, BioLegend), F4/80-PE (0.25 µg/ml, clone BM8, ThermoFisher scientific), CD43 BUV737 (1 mg/ml, clone S7, BD Bioscience), Ly6C-APC (1 µg/ml, clone HK1.4, ThermoFisher scientific), CD11b-BV421 (2 µg/ml, clone M1/70, BioLegend), Ly6G AF700 (1 µg/ml, clone 1A8, Biolegend) and MHCII-PerCP-Cy5.5 (2 µg/ml, clone N418, BioLegend), CD206-Pe-Cy7 (5 µg/ml, clone C068C2, Biolegend), CCR_2_-BV711 (0.5 µg/ml, clone 475301, BD Bioscience), CD115-APC (2 µg/ml, clone AFS98, Biolegend). Cells were then washed, centrifuged (1 min, 300 *xg*), and resuspended in flow buffer containing 15 µl of CountBright™ Absolute Counting Beads (Thermofisher scientific) before being analysed. Samples were run using LSRFortessa cell analyser (BD Bioscience) and analysed using FlowJo software (v10.1, BD Bioscience). All gating strategies were generated by using Fluorescent Minus One (FMO) controls (Supplementary Fig. 1).

### 2.7 Immunohistochemistry of dorsal root ganglia

For immunohistochemistry of perfuse-fixed tissue, animals were *trans*-cardially perfused with saline solution followed by 4 % paraformaldehyde (PFA, VWR chemicals) in PBS before L3, L4 and L5 DRGs were excised. Transverse DRG sections (10 µm) were then cut on a cryostat (Bright instruments) and mounted onto Superfrost Plus microscope slides (ThermoFisher scientific). Sections were first permeabilised with PBS containing 0.1 % Triton-X-100 (PBS-T; Sigma-Aldrich) for 15 min, then blocked with 3 % BSA (Sigma-Aldrich) for 1 h and finally incubated overnight with goat anti-rabbit CGRP (2 μg/ml, Cell Signalling), followed by anti-rabbit Alexa Flour 594 secondary antibody (1 μg/ml, Invitrogen) for 1 h. Slides are then washed 3 times for 5 min in 0.1 % PBS-T and mounted in DAPI containing mounting solution (Invitrogen). All antibodies were prepared in 0.1 M PBS with 0.1 % BSA and 0.1 % Triton X-100 (Sigma). For negative controls, the primary antibody was omitted; this resulted in the absence of staining. Images for immunofluorescence analysis were captured using a Zeiss LSM400 fluorescence microscope and analysed using ImageJ software (1.50i, Wayne Rasband, National Institutes of Health, USA). CGRP^+^ profiles, indicative of number of CGRP positive neuron cells, were quantified within fixed areas (4 × 10^4^ µm) per section. At least four sections from three mice per group were analysed.

### 2.8 Primary culture of BMDMs, treatments and flow Cytometry.

Hematopoietic stem cells were harvested from the femur and tibia bone marrow of mice. Bone marrow cells were differentiated into macrophages by culturing for 7 days at 37^◦^C and 5 % CO_2_ in 8 ml high glucose Dulbecco’s modified eagle media (DMEM) supplemented with 10 % heat-inactivated fetal bovine serum (FBS, Gibco), 1 % penicillin/streptomycin (P/S) and 10 % supernatant derived from L929 fibroblasts (L929-condition media) as a source of macrophage colony-stimulating factor (Englen et al., 1995) in 100 mm non-tissue culture treated Petri dishes (ThermoFisher Scientific). On day 5, 3 ml of medium was removed and an additional 5 ml of medium was added. Gentle scrapping was used to lift cells off dish surface. Cells were then counted and resuspended in DMEM at the concentration of 1x10^6^ per well of a 6 well plate.

Cells were incubated for 6 or 24 h with either 200 ng/ml FKN (RD Systems), 100 ng/ml LPS (Invitrogen) or PBS control. After treatment, supernatant were stored for further analysis and cells were treated with anti-mouse CD16/CD32 antibody (Clone 2.4G2, BD Biosciences, San José, CA) and stained with the following antibodies: CD45.1-BV786 (5 µg/ml, clone 30-F11, BioLegend), F4/80-PE (0.25 µg/ml, clone BM8, ThermoFisher scientific), Ly6C-APC (1 µg/ml, clone HK1.4, ThermoFisher scientific) CD11b-BV421 (2 µg/ml, clone M1/70, BioLegend), and MHCII-PerCP-Cy5.5 (2 µg/ml, clone N418, BioLegend), CD206-Pe-Cy7 (5 µg/ml, clone C068C2, Biolegend), CCR_2_-BV711 (0.5 µg/ml, clone 475301, BD Bioscience). Stained cells were acquired on an LSR Fortessa cytometer. All gating strategies were generated using Fluorescent Minus One (FMO) controls (Supplementary Fig. 1).

### 2.9 HUVEC cell culture, treatments, and analysis.

HUVEC were cultured in T75 flasks coated with 1 % gelatin (Sigma) in Endothelial Cell Growth Media (PromoCell) and used up to passage 6. Cells were grown to confluence in 6 well plate coated with 0.5 % gelatin and incubated overnight with CGRP (1 μM, RD System), in presence or absence of CGRP antagonist CGRP 8–37 (100 μM, RD System) in Endothelial Cell Growth Media (PromoCell). Following treatment with an Fc receptor blocking solution, cells were fixed, permeabilised with eBioscience Fix/perm solutions and stained with anti-ICAM-1-PE (5 μg/ml, clone HA58, Biolegend), anti-VCAM-1-BV711 (2 μg/ml, clone 5110C9, BD Optibuild) and anti FKN-APC (3 μg/ml, clone 51637, Biotechne) antibodies. Cells were analysed using LSR Fortessa cytometer and at least 10,000 events were acquired.

For immunocytochemistry, 1x10^4^ cells were plated on 0.5 % gelatin coated coverslips and after treatments as described above, cells were washed and fixed in cold 4 % PFA (4 °C, 30 min). After fixation, cells were washed with PBS and then blocked in PBS with 0.1 % Triton and 0.2 % BSA (*T*-PBS; for intracellular staining) for 30 min at room temperature. Following blocking, HUVEC were incubated with primary specific antibodies against CD31 (5 μg/ml, Abcam) and anti FKN (10 μg/ml, Abcam) in *T*-PBS + 0.2 % BSA overnight at 4 °C. Cells were washed and incubated with secondary antibody Alexa Fluor 488 anti-rabbit (5 μg/ml, Molecular Probes Invitrogen) or Alexa Fluor 594 anti-mouse (5 μg/ml, Molecular Probes Invitrogen) in *T*-PBS + 0.2 % BSA for 1 h at 20 °C. Cells were then mounted on glass coverslips using Fluoroshield Histology Mounting Medium with DAPI (Sigma-Aldrich) and visualised under microscope (Zeiss LSM400 Imaging System).

### 2.10 Elisa

IL-6 concentration in BMDMs conditioned media and FKN concentration in centrifuged conditioned media of HUVEC were measured by enzyme immunoassay kit (Abcam) according to the manufacturers’ guidelines.

### 2.11 Western blot

BMDMs (2 × 10^6^) were incubated with FKN (200 ng/ml) at 37 °C for 5, 15, 30 and 60 min and with SB203580 (10 μM, RD System) or with GSK2606414 (100 nM, RD System) for 15 min prior to the 30 min incubation with FKN. Cells were lysed in 95 °C NuPAGE LSD sample buffer (Thermo Fisher Scientific) containing dithiothreitol (DTT 1 mM).

Samples were run on a 10 % w/v sodium dodecyl sulfate polyacrylamide tris–glycine gel (BioRad Cat# 4561035), transferred on PVDF membranes, and analysed by immunoblotting with the following antibodies: pP38 MAPK (Cell Signalling Technology, Cat# 9215), pERK-Thr202/204 (Cell Signalling Technology, Cat# 9101), P38 MAPK (Cell Signalling Technology, Cat#9212), ERK (Cell Signalling Technology, Cat#9102), β-Actin (ThermoFisher Scientific, Cat# AM4302).

HUVEC (1x10^6^) were incubated with CGRP (1 μM, R&D System) overnight, lysed as described above, and analysed by immunoblotting with the following antibodies: ADAM17 (Abcam, Cat#2051), β-Actin (ThermoFisher scientific, Cat# AM4302).

### 2.12 Statistical analysis

All statistical calculations were performed using GraphPad Prism 7 (GraphPad Software, Inc.). Data are shown as mean ± S.D. Differences between groups were identified by one-way ANOVA followed by Kruskal-Wallis, one-way ANOVA with Tukey or Bonferroni multiple-comparison tests, and two-way ANOVA with Tukey or Bonferroni multiple-comparison test.

## 3 Results

### 3.1 KBxN serum transfer- associated allodynia in CX_3_CR_1_ KO mice

**Fig. 1:**
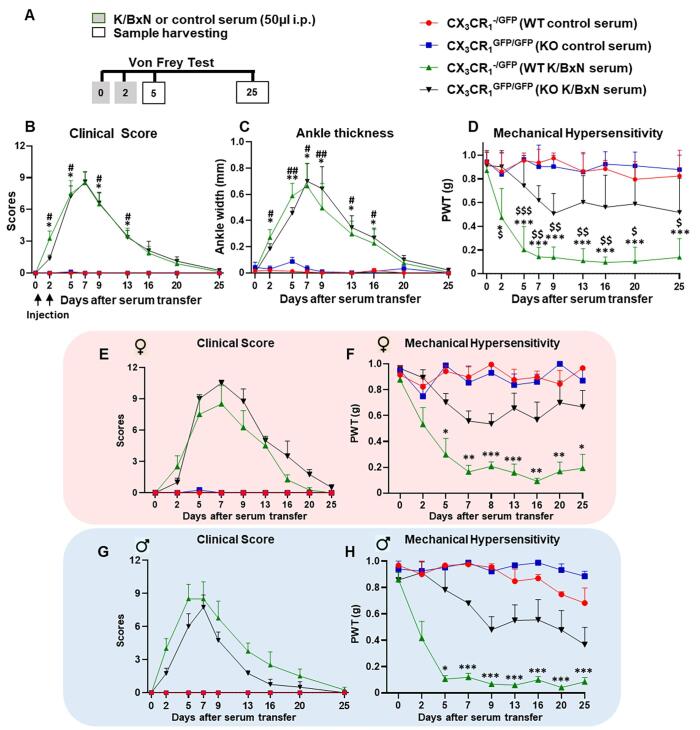
**K/BxN serum transfer is associated with allodynia in both female and male CX_3_CR_1_^+/GFP^ mice (WT) but not in CX_3_CR_1_^GFP/GFP^ mice (KO).** (A) K/BxN serum or control serum transfer experimental protocol (B) Hind paw clinical score in male and female mice. (C) Change in ankle thickness of male and female mice. (D) Mechanical hypersensitivity in male and female, PWT: Paw withdrawal thresholds. (E) Females clinical score. (F) Males clinical score. (G) Mechanical hypersensitivity females. (H) Mechanical hypersensitivity males. Data are mean ± S.D.; n = 8 animals per group. *p< 0.05, **p< 0.01, ***p < 0.001 WT K/BxN vs same day WT control serum group, ^#^p<0.05, ^##^p<0.01, ^###^p<0.001 K/BxN KO vs same day KO control serum group, ^$^p<0.05, ^$$^p<0.01, ^$$$^p<0.001 K/BxN KO vs same day K/BxN WT two-way ANOVA, post hoc Tukey.

In the K/BxN model of inflammatory arthritis, we began our investigation on whether the FKN/CX_3_CR_1_ pair/signalling is involved in the development of nociception and/or joint swelling. We employed CX_3_CR_1_^+/GFP^ (used as WT) and CX_3_CR_1_^GFP/GFP^ (used as KO) littermate mice, where all monocyte, macrophage and microglia populations present green fluorescence, and assessed paw swelling (clinical scores and ankle thickness) and hind paw thresholds (mechanical hypersensitivity) following systemic administration of control serum and K/BxN serum (Fig. 1A). Control serum injections induced neither swelling nor mechanical hypersensitivity in WT and KO mice (Fig. 1 B-D). However, in WT mice, K/BxN serum transfer resulted in paw swelling which peaked at days 5–7 and resolved by days 20–25 (Fig. 1 B,C). Hind paw mechanical hypersensitivity (allodynia) developed in association with paw swelling (days 2–7) but was present also when swelling had significantly resolved (day 16–25) (Fig. 1D). In KO mice, K/BxN serum transfer produced paw swelling as in WT, whereas allodynia was significantly attenuated in comparison to WT over the whole time course, from day 2 to day 25 (Fig. 1 B-D). Notably, clinical scores, and mechanical allodynia were detected in both male and female WT and attenuation of allodynia was found in KO of both sexes (Fig. 1 E-H).

Since the CX_3_CR_1_ transgenic mouse is a global KO and CX_3_CR_1_ receptor is expressed by monocytes, macrophages, but also microglia, we examined microglia activation in the dorsal horn of the spinal cord of K/BxN WT and KO. At day 5 and day 25 after K/BxN serum transfer, we observed significant microgliosis quantified as increased number of CD45^low^CD11b^+^GFP^+^ cells (flow cytometry) and Iba1^+^ cells (immunohistochemistry) (Supplementary Fig. 2 A-C) and microglial activation (monitored by quantification of p-p38 MAPK in Iba1^+^ cells) in both WT and KO animals (Supplementary Fig.2D), suggesting that CX_3_CR_1_ signalling does not regulate microglial response in this model of inflammatory arthritis.

Altogether, these behavioural data suggest that whilst CX_3_CR_1_ expression in monocytes/macrophages is not critical for development of paw swelling, confirming previous work (Jacobs et al., 2010, Wang et al., 2012), it is likely to contribute to the development and maintenance of allodynia in inflammatory arthritis. Therefore, in WT and KO mice, we quantified and assessed the phenotype of monocytes/macrophages in the hind paw, which is the area where nociceptive fibres innervate the joint, and in lumbar DRG which is the place for cell bodies of nociceptors.

### 3.2 Monocytes and macrophages in hind paws and lumbar DRG of CX_3_CR_1_ KO in K/BxN inflammatory arthritis

**Fig. 2:**
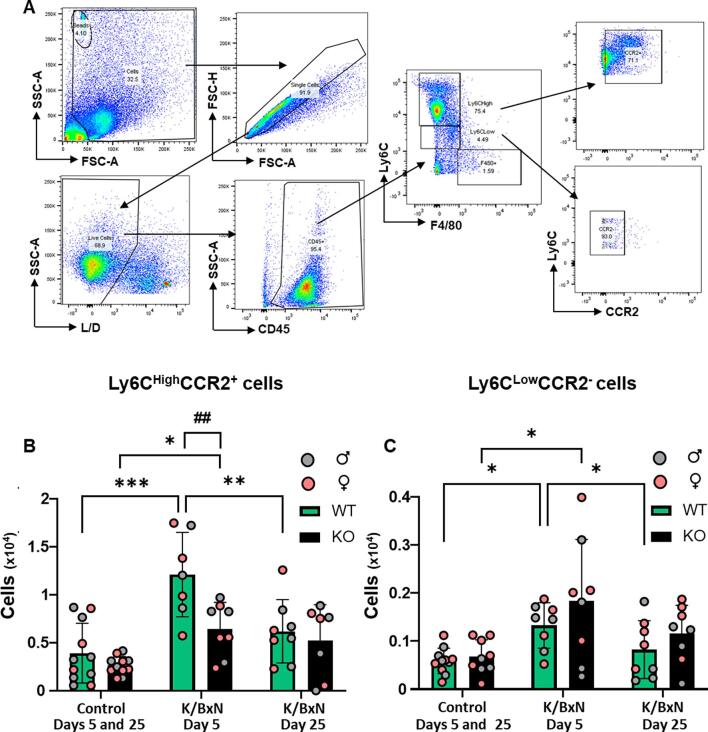
**Monocytes infiltrate WT and KO hind paws at day 5 following K/BxN serum transfer.** (A) Representative scatter plots of gating strategy for monocytes. Cells were first gated based on FSC-A and SCC-A, doublets were discriminated using FSC-A and FSC-H and viable cells using L/D dye and SSC-A. Non-classical monocytes were gated as CD45^+^F4/80^-^Ly6C^Low^CCR_2_^-^, while classical monocytes were CD45^+^F4/80^-^Ly6C^High^CCR_2_^+^ (B, C) Bar charts present numbers of classical and non-classical monocytes. Data are mean ± S.D.; n = 8–13 animals per group. *p < 0.05, **p < 0.01, ***p < 0.05, same group, day comparisons, ^#^p < 0.05, ^##^ p < 0.01, ^###^p < 0.001 same day, group comparisons, two-way ANOVA with Tukey multiple-comparison test.

To study dynamics of monocyte influxes, we monitored GFP^+^ cells, together with lineage markers Ly6C and CCR_2_ which identify classical monocytes (Fig. 2A). In WT hind paw cell suspension, we measured a significant increase of Ly6C^high^ cells at day 5, but not day 25 after K/BxN serum transfer in comparison to control serum (Fig. 2B). Infiltrating monocytes were Ly6C^high^CCR_2_^+^ but also Ly6C^low^CCR_2_^-^ (Fig. 2 B,C). Similarly in KO paw cell suspension, Ly6C^high^ cells were found at day 5 but not day 25 after K/BxN serum transfer (Fig. 2B) and both Ly6C^high^CCR_2_^+^and Ly6C^low^CCR_2_^-^ cells were significantly higher than in control serum cell suspension (Fig. 2 B,C). At day 5 after K/BxN serum transfer Ly6C^high^CCR_2_^+^ cells were lower in KO than in WT (Fig. 2B). At day 5 and 25 after K/BxN serum transfer female and male hind paws showed similar infiltration of monocytes with no difference between the sexes.

**Fig. 3:**
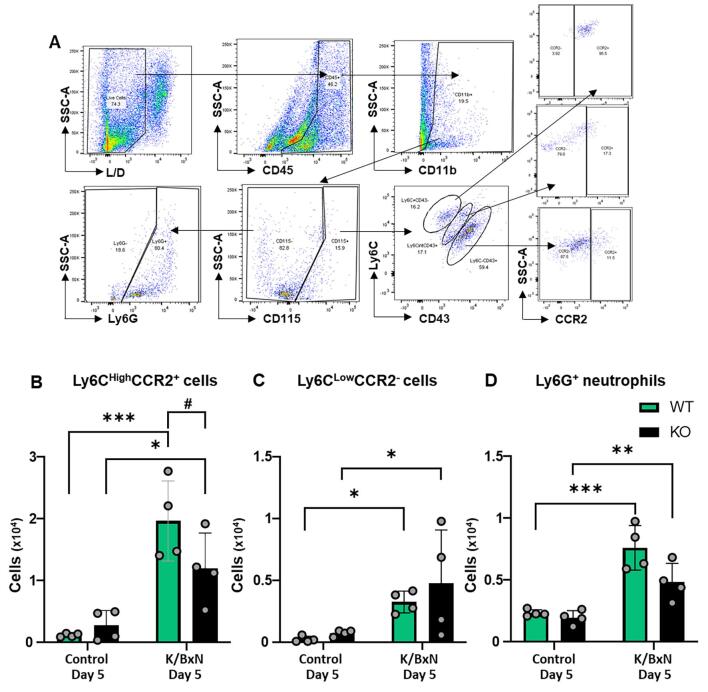
**Classical and non-classical monocytes and neutrophils infiltrate WT and KO hind paws at day 5 following K/BxN serum transfer.** (A) Scatter plots of gating strategy for monocytes and neutrophils. Viable cells were gated using L/D dye and SSC-A: non-classical monocytes were identified as CD45^+^CD11b^+^CD115^+^Ly6C^low^CD43^+^CCR2^-^, classical monocytes as CD45^+^CD11b^+^CD115^+^Ly6C^high^CD43^-^CCR2^+^ and neutrophils as CD45^+^CD11b^+^CD115^-^Ly6G^+^. (B, C, D) Bar charts present number of classical monocytes, non-classical monocytes and neutrophils in WT and KO hind paws. Data are mean ± S.D.; n = 4 animals per group. *p < 0.05, **p < 0.01, ***p < 0.05, same group, day comparisons ^#^p < 0.05, ^##^p < 0.01, ^###^p < 0.001 same day, group comparisons, two-way ANOVA with Tukey multiple-comparison test.

Since monocyte influx in the hind paw was noticeable in males and females at day 5 after K/BxN serum transfer and given that this model is associated with neutrophil infiltration (Norling et al., 2016), we investigated further the presence of both cell types in WT and KO in male mice. Monocytes were first identified as CD45^+^CD11b^+^CD115^+^ and then further subdivided in classical (Ly6C^high^CD43^-^CCR2^+^), non-classical (Ly6C^low^CD43^+^CCR2^-^), while neutrophils were identified as CD45^+^CD11b^+^CD115^-^Ly6G^+^ events (Fig. 3A). This gating strategy confirmed presence of both classical and non-classical monocytes in WT and KO compared to control paws (Fig. 3 B,C) with lower number of classical monocyte in KO compared to WT (Fig. 3B). Neutrophil numbers were higher in both WT and KO after K/BxN serum transfer compared to control serum (Fig. 3C), as expected since neutrophils do not express CX_3_CR_1_ (Jung et al., 2000).

**Fig. 4:**
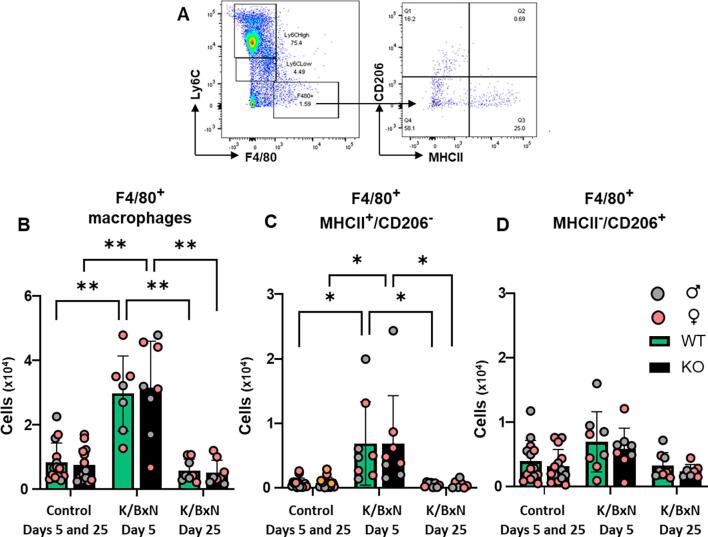
**Macrophage numbers increase in WT and KO hind paws at day 5 following K/BxN serum transfer** (A) Representative scatter plots of the gating strategy for macrophages CD45^+^Ly6C^-^F4/80^+^ which were further analysed for M1-like (F4/80^+^MHCII^+^) and M2-like (F4/80^+^CD206^+^) phenotypes. (B, C, D) Bar charts present numbers of macrophages (CD45^+^F4/80^+^), M1-like macrophages (F4/80^+^MHCII^+^) and M2-like macrophages (F4/80^+^CD206^+^) in WT and KO at day 5 and day 25 after K/BxN serum transfer. Data are mean ± S.D.; n = 8–13 animals per group. *p < 0.05, **p < 0.01, ***p < 0.001 same group, day comparisons, two-way ANOVA with Tukey multiple-comparison test.

Next, we quantified macrophages which were identified as CD45^+^, Ly6C^-^ and F4/80^+^ events, prior to characterization through MHCII (M1-like) and CD206 (M2-like) measurements (Fig. 4A). In WT hind paw cell suspensions, F4/80^+^ cells (macrophages) were higher than controls at day 5 but not day 25 after K/BxN serum transfer (Fig. 4B). At day 5, MHCII^+^ CD206^-^ (M1-like) macrophages were higher in K/BxN than controls whilst MHCII^-^CD206^+^ (M2-like) cell numbers were comparable (Fig. 4 C,D). Likewise, in KO hind paw homogenates F4/80^+^ cells were higher than controls at day 5 and not day 25 after K/BxN serum transfer (Fig. 4B) with MHCII^+^ CD206^-^ (M1-like) higher in K/BxN than controls (Fig. 4C) and MHCII^-^CD206^+^ (M2-like) cell numbers comparable between control and K/BxN paw homogenates (Fig. 4D). Macrophage numbers and phenotype were comparable in WT and KO under both control serum and K/BxN conditions in males and females. (Fig. 4 B-D).

Altogether, these analyses in hind paws, support existing evidence (Brunet et al., 2016, Misharin et al., 2014) that at peak of paw swelling associated with allodynia, both classical and non-classical monocytes infiltrate the hind paw and macrophages display a pro-inflammatory phenotype (Supplementary Fig. 3). Moreover, results in KO mice indicate that CX_3_CR_1_ expression in monocytes is associated with reduced infiltration of classical monocyte which correlates with the less severe allodynia in KO at 5 K/BxN serum transfer and may contribute to sensitization of nociceptive fibre terminals in arthritis joints. Nevertheless, CX_3_CR_1_ expression in macrophages is unlikely to impact on number and phenotype of these cells in the hind paw.

**Fig. 5:**
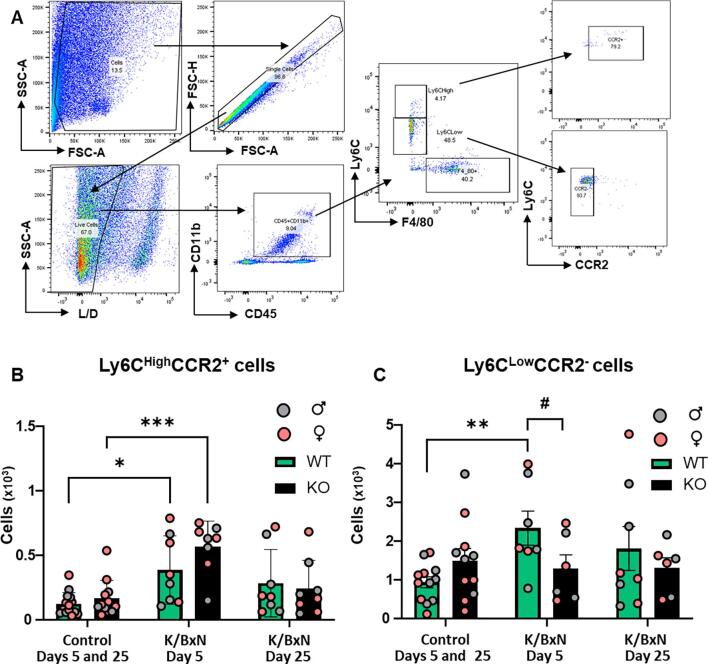
**Non-classical monocytes infiltrate WT, but not KO DRG at day 5 after K/BxN serum transfer.** (A) Representative scatter plots of the gating strategy for monocytes. Classical monocytes were CD45^+^CD11b^+^F4/80^-^Ly6C^High^CCR_2_^+^ cells, while non-classical were identified as CD45^+^CD11b^+^F4/80^-^Ly6C^Low^CCR_2_^-^ events. (B-C) Bar charts for classical and non-classical monocytes in WT and KO DRG. Data are mean ± S.D.; n = 7–13 animals per group. Data are mean ± S.D.; *p < 0.05, **p < 0.01, ***p < 0.001, same group, day comparisons, ^#^p < 0.05, ^##^p < 0.01, ^###^p < 0.001 same day, group comparisons, two-way ANOVA with Tukey multiple-comparison test.

Overall, our data point to a pro-nociceptive role for CX_3_CR_1_-expressing monocytes but not macrophages in arthritic joints. However, to step away from the site of primary inflammation, we moved our attention to the lumbar DRG which contain the cell bodies of nociceptive neurons that innervate the joint and performed flow cytometry analysis of monocytes and macrophages at day 5 and 25 after K/BxN serum transfer. In WT DRG, we observed that Ly6C cell (monocyte) numbers were higher at day 5 but not day 25 after K/BxN serum compared to control serum (Fig. 5 A-C). These cells were predominantly Ly6C^low^CCR_2_^-^ with a small percentage (≤10 %) being Ly6C^high^CCR_2_^+^ (Fig. 5 B,C). In KO DRG, Ly6C^high^CCR_2_^+^ cell numbers were higher in K/BxN serum compared to controls at day 5 (Fig. 5B). However, Ly6C^low^ CCR_2_^-^ cell numbers in K/BxN serum transfer DRG were lower than in WT (Fig. 5C). Overall, these analyses of DRG data suggest that CX_3_CR_1_ expressing monocytes (Ly6C^low^CCR_2_^-^) infiltrate the DRG at day 5 after K/BxN serum transfer, and absence of this chemokine receptor impairs such an infiltration (Supplementary Fig. 4A).

**Fig. 6:**
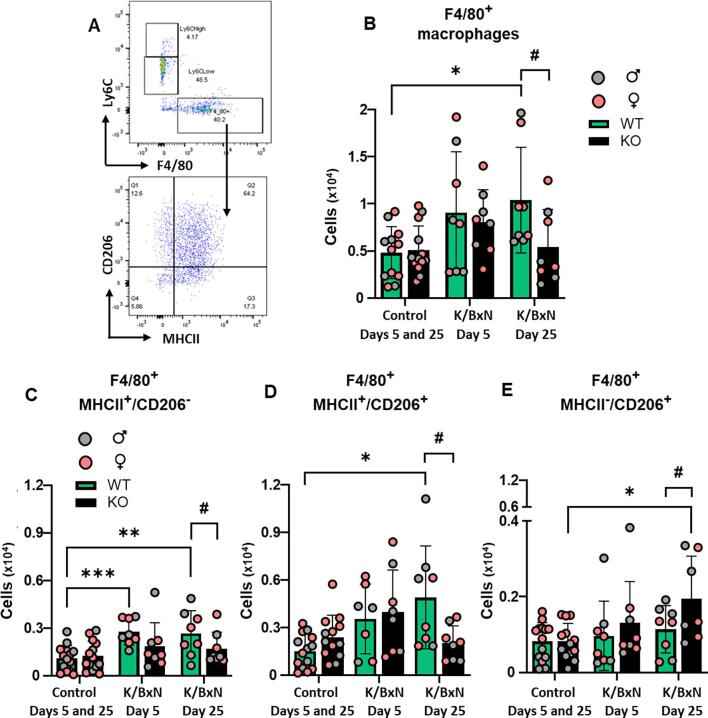
**K/BxN serum transfer is associated with increase in proinflammatory macrophages recruitment in DRG of WT but not KO.** (A) Representative scatter plots of immune cells sorted from lumbar DRG dissected at day 25 after transfer of control serum and K/BxN serum. Macrophages (CD45^+^CD11b^+^F4/80^+^Ly6C^-^) cells were stained for CD206 and MHCII to quantify proportion of M1-like (CD206^-^MHCII^+^), M1/M2-like (CD206^+^MHCII^+^) and M2-like (CD206^+^MHCII^-^) phenotypes. (B) Bar charts present numbers of DRG macrophages (F4/80^+^) at day 5 and day 25 after K/BxN serum transfer in WT and KO. (C-E) Bar charts present numbers of M1-like macrophages (F4/80^+^MHCII^+^CD206^-^), M1/M2-like macrophages (F4/80^+^MHCII^+^CD206^+^) and M2-like macrophages (F4/80^+^MHCII^-^CD206^+^) in WT and KO. Data are mean ± S.D.; n = 7–13 animals per group. Data are mean ± S.D.; *p < 0.05, **p < 0.01, ***p < 0.001, same group, day comparisons ^#^p < 0.05, ^##^p < 0.01, ^###^p < 0.001 same day, group comparisons, two-way ANOVA with Tukey multiple-comparison test.

Next, we assessed numbers and phenotypes of macrophages in DRG. In WT DRG, we observed an increase of F4/80^+^ cells at day 5 which reached statistical significance at day 25 after K/BxN serum transfer compared to control serum (Fig. 6 A,B). Instead in KO DRG, at day 5 after K/BxN serum transfer the numbers of F4/80^+^ cells were comparable to both control serum and WT K/BxN, while at day 25 after K/BxN serum transfer, macrophage numbers were lower than in WT (Fig. 6B), indicating that in KO DRG lower infiltration of monocytes resulted in no increase of macrophages.

Then we characterised macrophage phenotypes. In WT DRG the number of MHCII^+^CD206^-^ cells (M1-like) were higher in K/BxN serum compared to control serum at both day 5 and day 25 (Fig. 6C), whereas MHCII^-^CD206^+^ cell numbers (M2-like) were unaltered at either day (Fig. 6E). Of interest, majority of F4/80^+^ cells were macrophages presenting both MHCII and CD206: these MHCII^+^CD206^+^ macrophages (∼80 %; M1/M2-like) were significantly increased at day 25 (Fig. 6D and Supplementary Fig. 5B). In KO DRG, neither MHCII^+^CD206^-^ cells nor MHCII^+^CD206^+^ macrophages were altered in K/BxN compared to control serum DRG (Fig. 6 C-E). However, at day 25, we detected decrease of MHCII^+^CD206^-^ and MHCII^+^CD206^+^ macrophages and increase of MHCII^-^CD206^+^ cells (M2-like) compared to WT (Fig. 6 C-E and Supplementary Fig. 5C).

As observed in hind paws, monocyte and macrophage numbers were similar in male and female DRG not only under control condition, but also at day 5 and day 25 after K/BxN serum transfer (Fig. 5,6).

Overall, these data indicate that in WT DRG at day 5 after K/BxN serum transfer, non-classical monocytes and M1-like macrophages are the most abundant populations whereas at day 25 macrophages are mostly M1 and M1/M2-like (Supplementary Fig. 5D). Instead, in KO DRG at day 5 after K/BxN serum transfer the most notable populations are M1/M2-like and M2-like macrophages, and by day 25, M2-like macrophages are most prominent (Supplementary Fig. 5C).

Considering that M2-like macrophages are antinociceptive when injected intrathecally (Raoof et al., 2021, van der Vlist et al., 2022), their presence in the DRG correlates with the development of less severe allodynia after K/BxN serum transfer in CX_3_CR_1_ KO. Moreover, a further consideration based on WT data is that CX_3_CR_1_-expressing monocytes/macrophages in DRG contribute to nociceptive mechanisms in K/BxN inflammatory arthritis. Existing evidence, including ours, indicates that CX_3_CR_1_-expressing monocytes can bind to FKN, a transmembrane chemokine expressed by endothelial cells, to facilitate monocyte adhesion. Following cleavage of FKN chemokine domain by ADAM17, soluble FKN binding to CX_3_CR_1_ promotes monocytes transmigration through the endothelium (Landsman et al., 2009, Old et al., 2014, White and Greaves, 2012).

Therefore, our data suggest the possibility that in response to DRG sensory neuron activity triggered by peripheral inflammation, sFKN is liberated from the endothelium and promotes CX_3_CR_1_-monocyte adhesion and infiltration in the DRG parenchyma. Thus, we next investigated the mechanism which underlies sensory neuron-endothelium communication, and focussed on the neuropeptide CGRP, which is known to promote plasma extravasation in neurogenic inflammation (Chiu et al., 2012).

### 3.3 Sensory neuron CGRP in K/BxN inflammatory arthritis

**Fig. 7:**
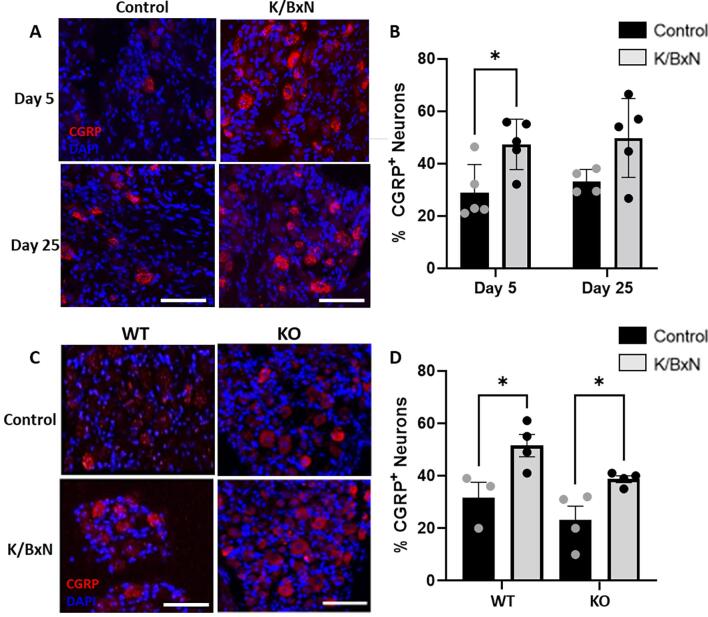
**CGRP expression is upregulated in lumbar DRG neurons at peak joint swelling 5 days after K/BxN serum transfer.** (A) Representative images of lumbar DRG neuron cell bodies displaying high CGRP- immunoreactivity (IR). (B) Quantification of percentage of DRG neuron cell bodies which express high CGRP-IR. (C) Representative images of WT and KO lumbar DRG neuron positive for CGRP- immunoreactivity (IR) at day 5 after K/BxN serum transfer. (D) Quantification of percentage CGRP positive neurons *p < 0.05 compared to same day control group, One-Way ANOVA, post-hoc Tukey. Data are expressed as mean ± S.D.; n = 3–5 mice per group. Scale bars, 50 µm.

Sensory neuron peptide content undergoes significant changes under peripheral inflammation conditions (Nieto et al., 2015, Staton et al., 2007). We found that CGRP expression was upregulated in DRG small and medium size neurons at day 5 but not 25 after K/BxN serum transfer compared to controls (Fig. 7 A,B). In both WT and KO DRG at day 5 after K/BxN transfer, CGRP expression was increased by ∼ 40 %, suggesting that CX_3_CR_1_ expression in monocytes/macrophages is unlikely to influence neuronal CGRP expression which is increased in sensory neurons under inflammatory arthritis.

**Fig. 8:**
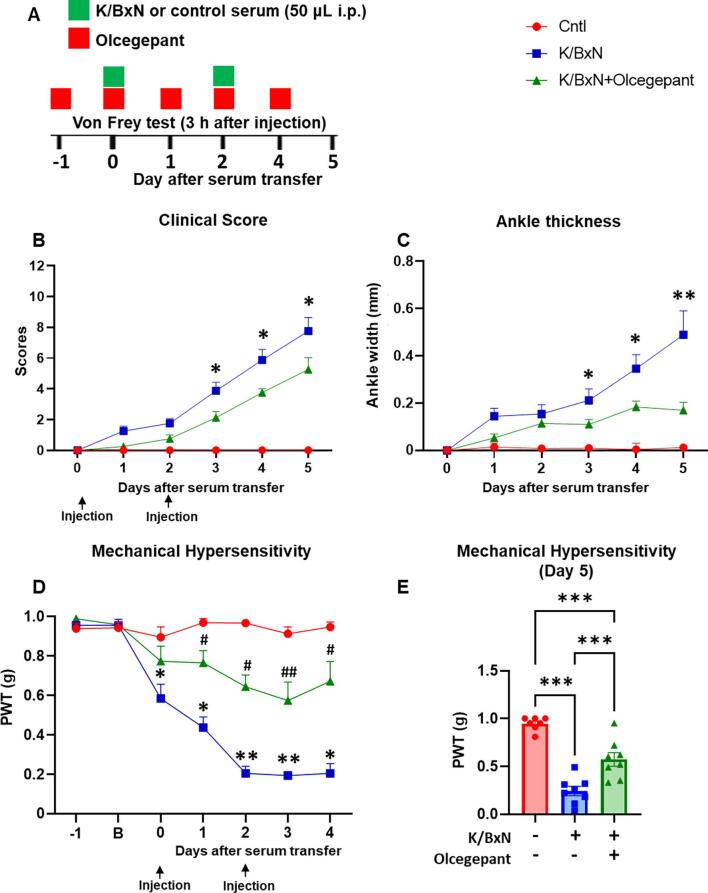
**Olcegepant treatment attenuates paw swelling and allodynia in K/BxN serum transfer inflammatory arthritis.** (A) K/BxN serum or control serum (50 μl) were administered at day 0 and day 2 to female mice. Olcegepant (1 mg/kg) or vehicle were administered intraperitoneally daily. (B) Clinical scores. (C) Change in ankle thickness. (D-E) Development of mechanical hypersensitivity. PWT: Paw withdrawal thresholds were monitored at 3 h after olcegepant injection. B: Baseline before K/BxN injection. Data are mean ± S.D.; n = 7–8 animals per group. *p < 0.05, **p < 0.01, ***p < 0.001 vehicle-K/BxN-group vs same day olcegepant-K/BxN-group. ^#^p < 0.05, ^##^p < 0.01 olcegepant-K/BxN-group vs same day vehicle-control serum, two-way ANOVA, post hoc Tukey. (E) ***p < 0.001, one-way ANOVA with Kruskal-Wallis test.

Since CGRP is released with activity by nociceptive neurons (Ebbinghaus et al., 2015) and promotes hind paw nociception and joint swelling, we tested the effect of systemic administration of the CGRP receptor antagonist, olcegepant, on swelling, allodynia and leukocyte accumulation in paws and DRG. We selected olcegepant, a small molecule antagonist, as this was recently shown to attenuate mechanical hypersensitivity in female mice in a model of neuropathic pain (Paige et al., 2022) and not to exert any effect on baseline pain responses in both male and female rodents (Christensen et al., 2019, Kopruszinski et al., 2021). Olcegepant administration (1 mg/kg daily for 5 days) to female mice (Fig. 8A), reduced clinical scores and ankle swelling at 4th injection on day 3 after K/BxN serum transfer, which is when swelling started to show, and this effect persisted for up to day 5 (Fig. 8 B,C). Olcegepant attenuated mechanical hypersensitivity from 3 h after the first injection of KBxN serum at day 0 for up to day 4 (Fig. 8D) and this anti-nociceptive effect was still detected at 24 h after the last administration at day 5 (Fig. 8E).

Altogether these behavioural data suggest that CGRP contributes to swelling and allodynia in inflammatory arthritis.

Next, we quantified the phenotype of monocyte/macrophages and neutrophils in hind paw cell suspensions and in lumbar DRG using the same markers as above (Fig. 3, Fig. 4, Fig. 5, Fig. 6).

**Fig. 9:**
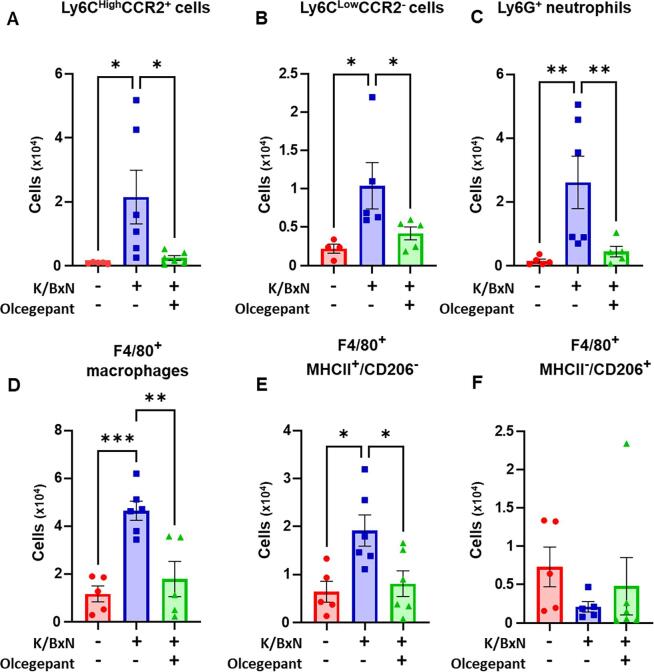
**Olcegepant treatment is associated with less leukocyte infiltration in the paw in K/BxN serum transfer inflammatory arthritis.** Within the paw cells suspension, classical monocytes were defined as CD45^+^CD11b^+^CD115^+^Ly6C^High^CD43^-^CCR2^+^ cells, non-classical as CD45^+^CD11b^+^CD115^+^F4/80^-^Ly6C^Low^CD43^+^CCR_2_^-^, while neutrophils were CD45^+^CD11b^+^CD115^-^Ly6G^+^. Macrophages were defined as CD45^+^CD11b^+^F4/80^+^Ly6C^-^ cells, with CD206 and MHCII staining used to define M1-like (CD206^-^MHCII^+^) and M2-like (CD206^+^MHCII^-^) phenotypes. (A-C) Bar charts present classical, non-classical monocytes and neutrophils in control serum, K/BxN serum, and K/BxN serum plus olcegepant paws. (D-F) Bar charts present total macrophages (F4/80^+^), M1-like macrophages (CD206^-^MHCII^+^), and M2-like macrophages (CD206^+^MHCII^-^) in the same groups. Data are mean ± S.D.; n = 7–8 animals per group. *p < 0.05, **p < 0.01, ***p < 0.001, one-way ANOVA with Kruskal-Wallis test.

In the paws, at day 5 after K/BxN serum transfer, olcegepant treatment resulted in fewer classical and non-classical monocyte, neutrophil and M−1 like macrophage numbers compared to vehicle group (Fig. 9A-F), indicating that CGRP drives generation of the inflammatory infiltrate within the arthritic paw.

**Fig. 10:**
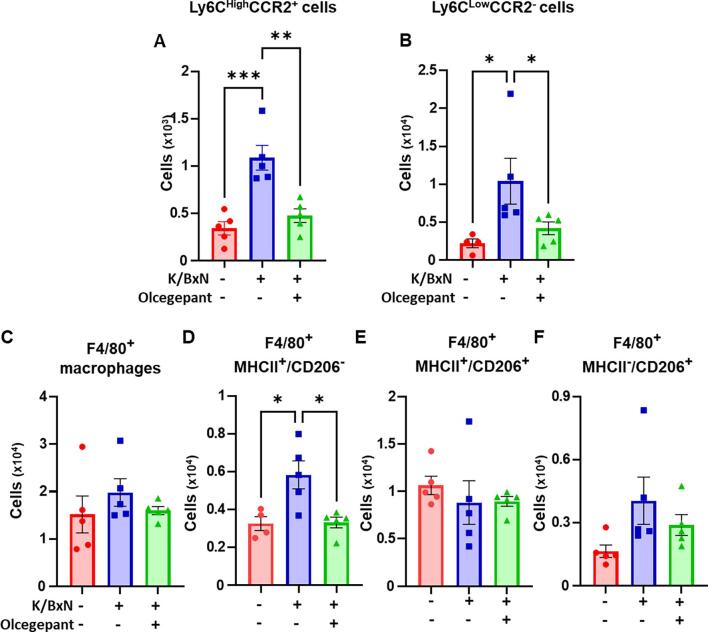
**Olcegepant treatment reduces monocyte and M1-like macrophage infiltration in the DRG of arthritic mice.** Classical monocytes were defined as CD45^+^CD11b^+^Ly6C^High^CD43^-^CCR2^+^ cells, non-classical as CD45^+^CD11b^+^F4/80^-^Ly6C^Low^ CD43^+^CCR_2_^-^, while neutrophils were CD45^+^CD11b^+^Ly6C^-^Ly6G^+^. Macrophages (CD45^+^CD11b^+^F4/80^+^Ly6C^-^) were stained for CD206 and MHCII labels were used to quantify M1-like (CD206^-^MHCII^+^), M1/M2-like (CD206^+^MHCII^+^) and M2-like (CD206^+^MHCII^-^) phenotypes. (A-B) Bar charts present numbers of classical, non-classical monocytes in control serum, K/BxN serum, and K/BxN serum plus olcegepant. (C-F) Numbers of total macrophages (F4/80^+^), M1-like macrophages (CD206^-^MHCII^+^), M1/M2 like macrophages (CD206^+^MHCII^+^) and M2-like macrophages (CD206^+^MHCII^-^) in the same groups. Data are mean ± S.D.; n = 7–8 animals per group. * p < 0.05, **p < 0.01, ***p < 0.001, one-way ANOVA with Kruskal-Wallis test.

When DRG were analysed for immune cells numbers and phenotype, we then observed that olcegepant treatment reduced Ly6C cells (both classical and non-classical monocytes) at day 5 after K/BxN serum transfer (Fig. 10A,B). In the macrophage analyses, olcegepant reduced MHCII^+^ (M1-like) macrophage recruitment, (Fig. 10D) without affecting either the M1/M2 or the M2 like macrophages (Fig. 10 E,F).

Overall, these data indicate that sensory neuron derived CGRP promotes infiltration of classical monocytes, non-classical monocytes, neutrophils, and macrophages in the hind paws as well as of classical monocytes, non-classical monocytes and M1 like macrophages in DRG.

Since these effects of CGRP would be concomitant to endothelial activation (Crossman et al., 1990), and CX_3_CR_1_ expressing monocytes infiltrate the DRG at day 5 after K/BxN serum transfer, we postulated that CGRP may be critical for the liberation of sFKN from the endothelium which would then attract monocytes.

### 3.4 Liberation of endothelial fractalkine by sensory neuron CGRP

**Fig. 11:**
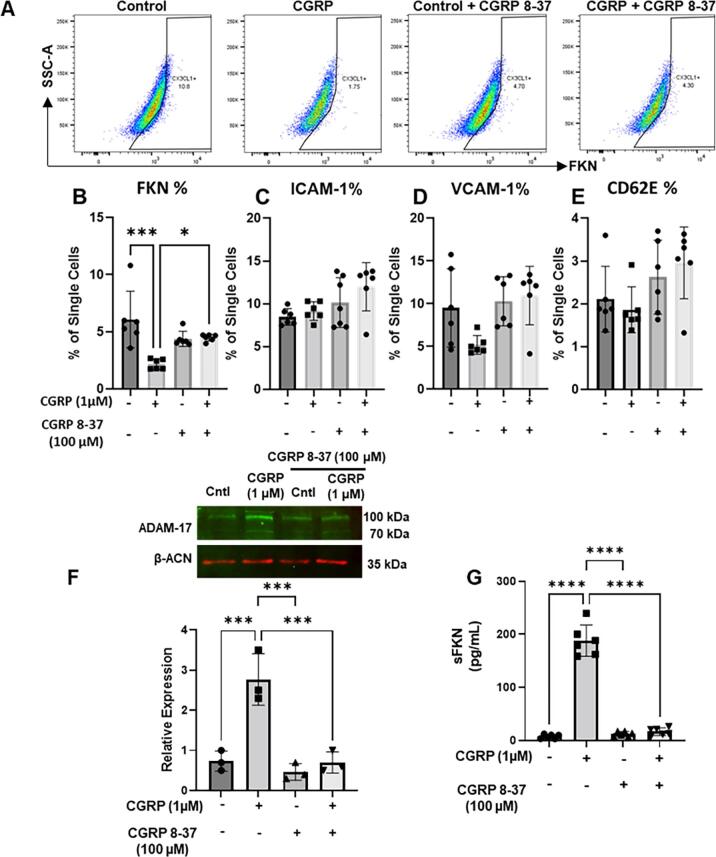
**CGRP induces release of endothelial FKN via ADAM-17 activation.** HUVEC treated with vehicle, CGRP (1 μM), CGRP 8–37 (100 μM) or the combination of CGRP and CGRP 8–37 were analysed by flow cytometry and Western blot. Conditioned media was used for FKN release. (A) Representative scatter plots of staining for FKN in HUVEC treated with PBS (control), CGRP, CGRP 8–37 and combination of CGRP and CGRP 8–37. Bar charts present % of HUVEC positive for FKN (B), ICAM-1 (C), VCAM-1 (D), CD62E (E) staining. Data are means ± S.D., n = 5 for each group. *p < 0.05, **p < 0.01, ***p < 0.001 one-way ANOVA, post hoc Bonferroni. (F) Representative Western blot and quantification of ADAM-17 protein levels induced by CGRP in HUVEC. Data are means ± S.D., n = 3 for each group. *p < 0.05, **p < 0.01, ***p < 0.001 one-way ANOVA, post hoc Bonferroni. (G) FKN levels in HUVEC supernatant measured by ELISA. Data are mean ± S.D., n = 6 for each group. *p < 0.05, **p < 0.01, ***p < 0.001 one-way ANOVA, post hoc Bonferroni.

To test this hypothesis, we evaluated the effect of CGRP incubation with HUVEC cells on the expression of FKN and selected adhesion molecules using flow cytometry. Incubation of HUVECs with CGRP (1 µM overnight) resulted in a significant decrease of membrane-bound FKN expression both when expressed as % of single cells and MFI units (Fig. 11 A,B and Supplementary Fig. 5) and this effect was reversed by the CGRP receptor antagonist CGRP 8–37 (Fig. 11 A,B, Supplementary Fig. 5). The effect of CGRP was specific as it did not alter expressions of ICAM-1, VCAM-1 and CD62E (Fig. 11 C-E, Supplementary Fig. 5). Since membrane-bound FKN is cleaved into sFKN by the protease ADAM-17 which is also expressed by endothelial cells (Gooz et al., 2009), we quantified ADAM-17 expression and release of sFKN after CGRP application to HUVECs. We observed that CGRP application resulted in up-regulation of ADAM-17, and this effect was blocked by CGRP 8–37 (Fig. 11F). Furthermore, under the same conditions CGRP increased sFKN content in the incubation media and CGRP 8–37 blocked this effect (Fig. 11G). Finally, using immunohistochemistry we could visualise that CGRP incubation reduced expression of membrane-bound FKN expression in HUVEC (CD31^+^ cells) and this effect was inhibited by CGRP 8–37 (Supplementary Fig. 5).

These *in-vitro* data indicate that in endothelial cells CGRP upregulates ADAM-17 which liberates sFKN and suggest that in K/BxN DRG, CGRP released with activity by nociceptive neurons would result in liberation of endothelial sFKN that can activate CX_3_CR_1_ receptor in monocytes and promotes monocyte infiltration in the DRG parenchyma. In our final set of experiments with bone marrow-derived macrophages, we identified the mechanism by which sFKN activation of CX_3_CR_1_ promotes nociceptive signalling.

### 3.5 Fractalkine modulates macrophage phenotype towards M1 and induces release of IL-6

**Fig. 12:**
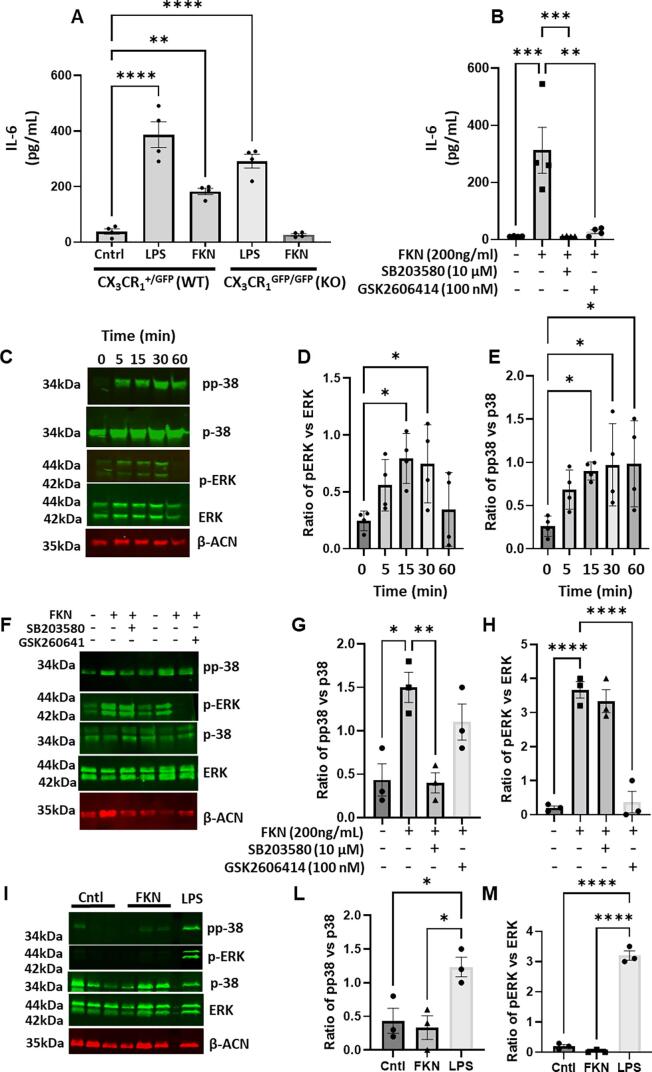
**FKN induces release of IL-6 via phosphorylation of ERK and p38 in BMDMs.** BMDMs from WT (CX_3_CR_1_^+/GFP^) or KO (CX_3_CR_1_^GFP/GFP^) mice were treated with 200 ng/ml of FKN for up to 6 h and IL-6, phosphorylation of ERK and p38 monitored by ELISA and Western blot, respectively. (A) Quantification of IL-6 in supernatants of WT and KO BMDMs treated with LPS and FKN. (B) Quantification of IL-6 in supernatants of WT BMDMs treated with FKN in presence of pERK or pp38 inhibitor (GSK2606414 and SB203580 respectively). (C-E) Representative Western blot and quantification of p-p38 and p-ERK protein levels induced by FKN in WT BMDMs after 5, 15, 30 and 60 min. (F-H) Representative Western blot and quantification of p-p38 and p-ERK protein levels induced by FKN in WT BMDMs after 30 min in presence of pERK or pp38 inhibitor (GSK2606414 and SB203580 respectively). (I-M) Representative Western blot and quantification of p-p38 and p-ERK protein levels induced by FKN in KO BMDMs after 30 min. Data are mean ± S.D.; n = 4 for each group. *p < 0.05, **p < 0.01, ***p < 0.001, p****< 0.0001 one-way ANOVA, post hoc Bonferroni.

Since monocyte/macrophage invasion in K/BxN DRG is associated with behavioural allodynia in WT and attenuation of allodynia in CX_3_CR_1_ KO mice, we evaluated the effect of FKN on pro-nociceptive cytokine release in bone marrow derived macrophages (BMDM). Our preliminary data in differentiated THP-1 cells indicated that IL-6 and IL-8 were up-regulated by FKN in macrophages more than IL-1 and TNF. Thus, we have only considered IL-6 in this study. We observed that FKN (200 ng/ml) induced significant release of IL-6 in WT, but not in CX_3_CR_1_ KO BMDM (Fig. 12 A,B), and in WT BMDM, incubation of FKN promoted phosphorylation of p38 MAPK and ERK (Fig. 12 C-E). Both IL-6 release and phosphorylation of p38 MAPK and ERK induced by FKN were significantly reduced when BMDMs were treated pERK or pp38 inhibitors (GSK2606414 and SB203580, Fig. 12 B, F-H). Moreover, KO BMDMs stimulated with FKN showed neither pERK, nor p38 phosphorylation (Fig. 12 I-M).

**Fig. 13:**
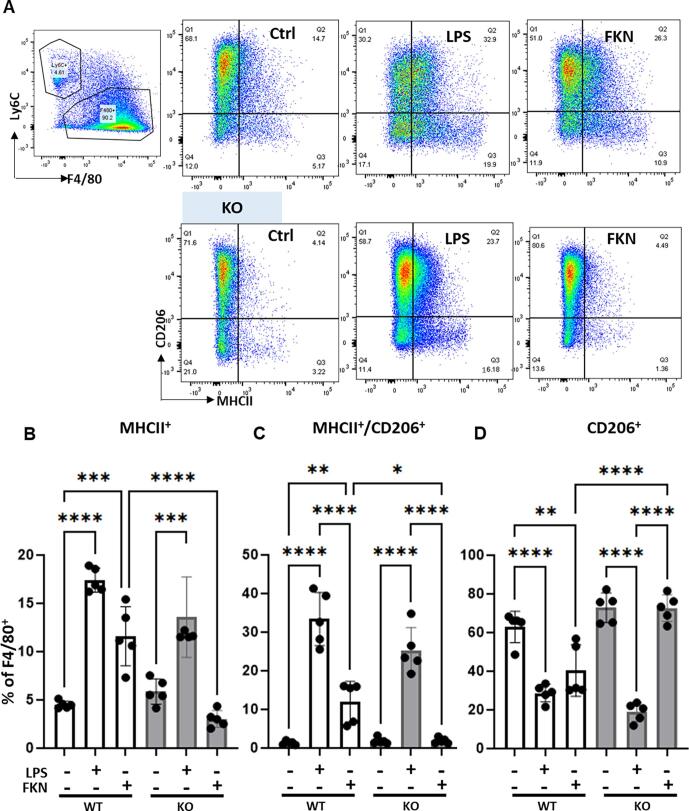
**FKN polarises macrophages towards a pro-inflammatory phenotype.** BMDMs from WT (CX_3_CR_1_^+/GFP^) and KO (CX_3_CR_1_^GFP/GFP^) mice were treated with FKN (200 ng/ml) or LPS (100 ng/ml) for 24 h and CD206 and MHCII markers analysed by flow cytometry. (A) Representative scatter plots of macrophages incubated with vehicle, LPS or FKN 24 h. Macrophages (CD45^+^CD11b^+^F4/80^+^Ly6C^-^) were stained for CD206 and MHCII to define M1-like (CD206^−^MHCII^+^), M1/M2-like (CD206^+^MHCII^+^) and M2-like (CD206^+^MHCII^−^) phenotypes. (B-D) Bar charts present numbers of M1-like macrophages, M1/M2-like macrophages, and M2-like macrophage. One-way ANOVA, post hoc Bonferroni. Data are mean ± S.D.; n = 5 for each group. *p < 0.05 and **p < 0.01, ***p < 0.001, p****< 0.0001, one-way ANOVA, post hoc Bonferroni.

Furthermore, incubation of WT, but not KO BMDMs with FKN for 24 h resulted in higher percentage of F4/80^+^ cells expressing MHCII (M1-like) and MHCII/CD206 (M1/M2 -like) compared to controls (Fig. 13 A-C). CD206^+^ cells (M2-like) were no different from controls after FKN incubation and were significantly reduced after 24 h incubation with LPS (Fig. 13D).

These *in-vitro* data indicate that in macrophages FKN results in activation of intracellular kinases, polarisation towards an M1-like phenotype and release of the pro-nociceptive cytokine IL-6.

## 4 Discussion

In this study we show that in the K/BxN serum transfer model of inflammatory arthritis, acute arthritic scores (swelling) and allodynia (mechanical hypersensitivity) are associated with invasion of monocytes and presence of M1-like macrophages in the joints and DRG. When K/BxN inflammatory arthritis resolve, macrophages return to basal levels and display M2-like phenotype in joints. However, allodynia persists and correlates with the presence of numerous M1-like macrophages in DRG. Furthermore, absence of CX_3_CR_1_ receptor, expressed by monocytes/macrophages, results in significant attenuation of allodynia, monocyte recruitment in joints and DRG and M1-like macrophage infiltrate in DRG. These findings expand on the assumption that monocytes/macrophages in DRG are relevant to nociceptive mechanisms and suggest that CX_3_CR_1_ receptor plays a significant role in the initiation and persistence of allodynia in inflammatory arthritis. Microglial CX3CR1 receptors appear not to contribute to nociception in the KBxN model of RA. This is inconsistent with our data showing FKN-neutralising antibody reversal of nociception in the rat collagen-induced arthritis model of RA, which was associated with attenuation of microglial activation in the dorsal horn (Nieto et al., 2016). We speculate that active immunization in CIA versus passive immunization in K/BxN ST may be associated with distinct microglial pathways as for instance, microglial TLR-4 receptor plays a critical role in KBxN persistent allodynia (Christianson et al., 2011).

Under joint arthritis conditions, large numbers of monocytes infiltrate the joint and play critical roles in initiation, progression, and resolution of inflammation (Brunet et al., 2016, Misharin et al., 2014). Specifically, in K/BxN inflammatory arthritis, there is evidence that classical monocytes are critical for the progression of joint inflammation (Brunet et al., 2016). However, there is also proof that non-classical monocytes are crucial for the initiation of arthritis (Misharin et al., 2014). Nevertheless, severity of clinical scores is altered in neither CCR_2_ deficient nor CX_3_CR_1_ deficient mice (Jacobs et al., 2010, Wang et al., 2012) suggesting that neither chemokine receptors are critical for the onset and progression of joint swelling. We obtained similar findings and observed that CX_3_CR_1_ receptor deficiency affects neither severity of clinical scores, nor macrophage phenotype in arthritic paws though classical monocyte infiltration was reduced. Classical monocytes express CX_3_CR_1_ and CCR_2_ and we observed that deficiency of CX_3_CR_1_ results in infiltration of more CCR_2_^+^ monocytes in sciatic nerve in vincristine model of neuropathic pain (Montague et al., 2018). Nevertheless, a possible interaction in the K/BxN inflamed paw would have resulted in opposite results indicating that interactions of these chemokine receptors are tissue and model dependent.

Away from the joint in the DRG, we observed recruitment of non-classical and classical monocytes as well as presence of M1-like macrophages at peak inflammation at day 5 K/BxN arthritis. With the progression of arthritis, when joint inflammation resolves by day 25, DRG still present high numbers of M1-like macrophages. However, at this time point a good proportion (∼60 %) also express CD206 and as such are identified as dual M1/M2-like macrophages. Relevantly, deficiency of CX_3_CR_1_ receptor, impairs non-classical monocyte infiltration in DRG at day 5 K/BxN, and alters the macrophage dynamics; using our markers, in CX_3_CR_1_ KO DRG the phenotype of macrophages is mostly M1/M2-like at day 5 and M2 at day 25 K/BxN.

Taken together, these observations lead to two assumptions. The first assumption is that in inflammatory pain conditions, CX_3_CR_1_ receptors mediate monocyte/macrophage communication with sensory neurons responsible for nociceptive signalling. The second assumption is that the DRG are a preferable location over the joint for further delineation of neuro-immune mechanisms in nociceptive pain. Undeniably, inflamed joints would be a plausible site for such studies as they are innervated by primary afferents that i) transmit noxious signalling to the CNS, ii) can be sensitised by inflammatory mediators and iii) contribute to inflammation by releasing neuropeptides (neurogenic inflammation). However, our focus to the DRG is motivated by their location away from the joint since nociception is concomitant to paw inflammation but persists even when overt paw inflammation subsides.

Thus, we gathered evidence that the neuropeptide CGRP expression increases in sensory neurons in concomitance to joint inflammation, an observation which is in line with CGRP up-regulation in DRG in other models of arthritis and peripheral inflammation (Nieto et al., 2015, Seybold et al., 1995).

CGRP is a pro-nociceptive peptide that is released by the central terminals of primary afferent fibres in the dorsal horn of the spinal cord where it mediates mechanical hypersensitivity (Nieto et al., 2015). CGRP is also released at peripheral fibre terminals where it mediates neurogenic inflammation and regulates immune response to bacterial infection (Chiu et al., 2013, Chiu et al., 2012). We show that the small molecule CGRP antagonist, olcegepant prevents development of swelling and mechanical hypersensitivity in inflammatory arthritis and precludes monocyte and neutrophil infiltration in paws while reducing classical monocytes, non-classical monocytes and M1 like macrophage numbers in DRG. We suggest that sensory neuron-derived CGRP orchestrates inflammatory cell trafficking in inflammatory arthritis and corroborate this hypothesis with an *in-vitro* set of experiments. Herein, we show that CGRP induces the expression of endothelial ADAM-17 which liberates soluble FKN chemokine domain in HUVEC cells. Since FKN activation of CX_3_CR_1_ receptor induces M1-like phenotype in bone marrow-derived macrophages and release of pro-nociceptive cytokine IL-6, we suggest that in K/BxN DRG, neuronal CGRP activation of endothelial cells promotes monocyte infiltration via activation of endothelial FKN/monocyte CX_3_CR_1_ signalling which in turn sensitise neurons via downstream cytokine release (Supplementary Fig. 6).

Considering that the cell bodies of sensory neurons in DRG contribute to nociceptive mechanisms, and CX_3_CR_1_ KO develop less severe allodynia than WT, our data suggest that CX_3_CR_1_ receptors expressed in monocytes/macrophages play a critical role in nociceptive mechanisms in inflammatory arthritis.

A peculiarity and translational feature of the K/BxN serum transfer model of inflammatory arthritis is that whilst joint swelling resolves at 3–4 weeks after passive immunization, allodynia persists. We have recently proposed that DRG macrophages play a significant role in the maintenance of mechanical hypersensitivity in inflammatory arthritis (Allen et al., 2020). Here we add more prominence to these immune cells as in CX_3_CR_1_ KO DRG, macrophages display a predominant M2-like phenotype and allodynia is significantly attenuated in CX_3_CR_1_ KO mice. We suggest that the absence of CX_3_CR_1_ in monocytes/macrophages prevents macrophages from acquiring M1-like phenotype during progression of inflammatory arthritis by reducing the impact of endothelial FKN activation of CX_3_CR_1_ receptors in these cells. Since macrophage polarization between M1 and M2 states is a regulated phenomenon (Mounier et al., 2013, Sica and Mantovani, 2012), our data indicate that DRG macrophages are highly dynamic *in-vivo*. Under normal conditions, DRG macrophages distribute among 3 equally represented populations, namely M1-, M2- and M1/M2-like phenotypes, which suggests that M1 marker expression doesn’t rule out expression of M2 markers. At both day 5 and day 25 K/BxN, WT macrophages go through phenotypic transition to M1/M2-like macrophages, which provides evidence of polarization skewing. Importantly, polarization also occurs in CX_3_CR_1_ KO DRG, however the direction of skewing indicates preferred transition towards M2-like phenotype, which suggests a mechanistic role of CX_3_CR_1_ receptors in conferring M1-like phenotype in WT. This was partially recapitulated in our *in-vitro* studies where BMDMs treated with FKN acquired M1-like phenotype and at the same time supported by evidence that intraganglionic injection of FKN induces proinflammatory phenotype in macrophages (Kwon et al., 2015).

In conclusion, in inflammatory arthritis, sensory neuron activity in DRG promotes recruitment of monocytes via a CGRP-led mechanism that includes endothelial liberation of FKN and transmigration of CX_3_CR_1_-expressing monocytes. In DRG monocyte/macrophage CX_3_CR_1_ expression is likely to confer M1-like phenotype to macrophages and promote nociceptive neuron activation via release of factors, including IL-6 (see scheme in Supplementary Fig. 6). Thus, we suggest that peripheral CX_3_CR_1_ receptor offers a potential target to relieve arthritis pain.

## CRediT authorship contribution statement

**Silvia Oggero:** Conceptualization, Data curation, Formal analysis, Investigation, Methodology, Visualization, Writing – original draft, Writing – review & editing. **Chiara Cecconello:** Investigation. **Rita Silva:** Investigation. **Lynda Zeboudj:** Investigation, Methodology. **George Sideris-Lampretsas:** Investigation, Formal analysis. **Mauro Perretti:** Supervision, Writing – review & editing. **Marzia Malcangio:** Conceptualization, Formal analysis, Funding acquisition, Project administration, Resources, Supervision, Writing – original draft, Writing – review & editing.

## Declaration of Competing Interest

The authors declare that they have no known competing financial interests or personal relationships that could have appeared to influence the work reported in this paper.

## Data Availability

Data will be made available on request.
