# Supplementary material for: Dorsal root ganglia CX3CR1 expressing monocytes/macrophages contribute to arthritis pain
Source: Brain Behav Immun. 2022 Nov;106:289–306. doi: 10.1016/j.bbi.2022.09.008 (PMC10166715; doi:10.1016/j.bbi.2022.09.008)

**List of Supplementary Figures:**

**Supplementary Figure 1. Fluorescence Minus One Controls.**  Representative figures of Fluorescence Minus One control (FMO) of DRG leukocyte gating strategy (A), BMDMs gating strategy (B), Paw leukocyte gating strategy (C).

**
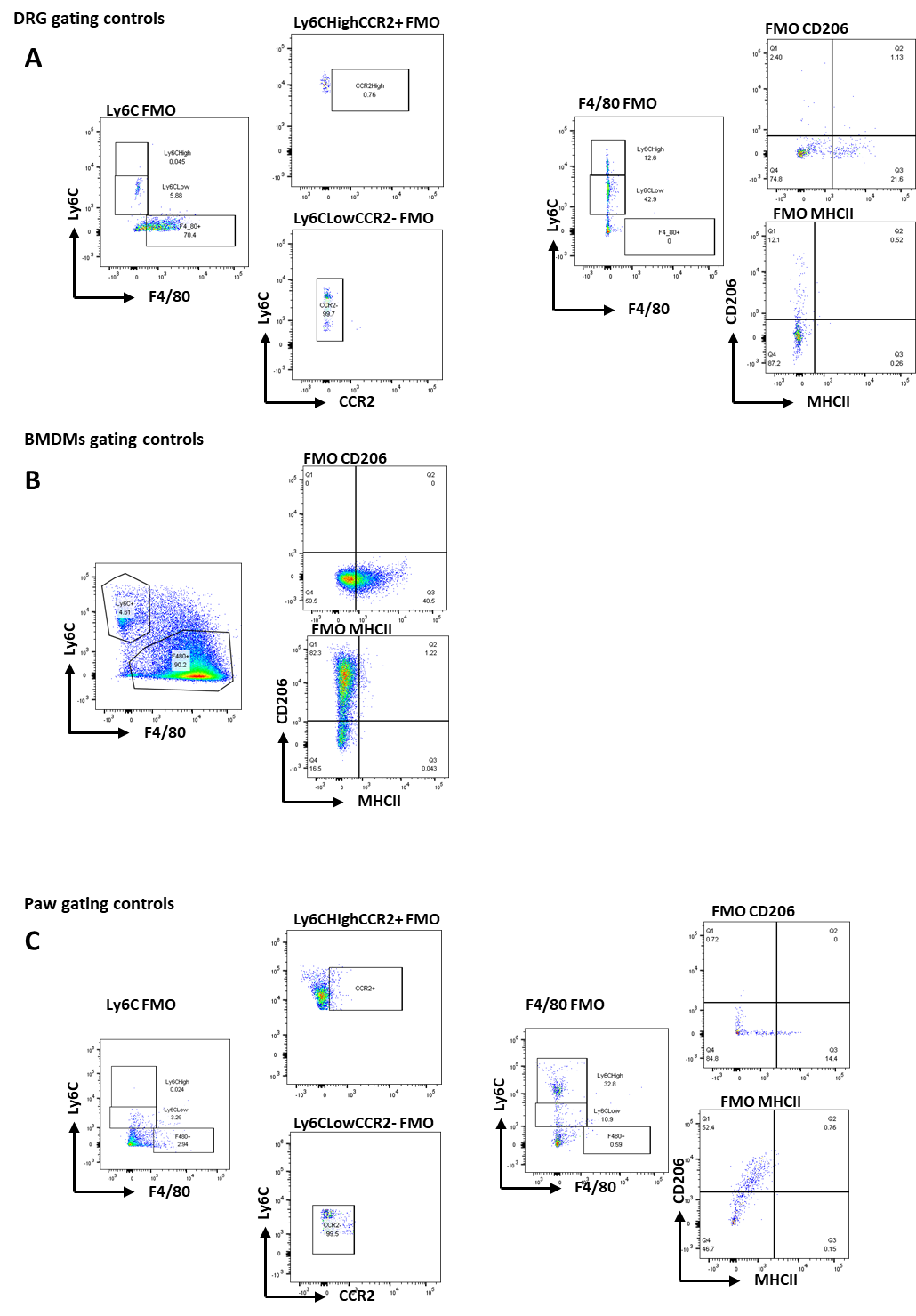
**

**Supplementary Figure 2. K/BxN serum transfer arthritis is associated with microglia activation in the spinal cord which is not altered in CX_3_CR_1_ KO.** (A) Scatter plots of gating strategy for microglial cells in male and female mice (GFP^+^ cells). (B) Bar charts present numbers of microglia cells in WT and KO dorsal horns at day 5 and 25 after K/BxN serum transfer. (C) Quantification of Iba^+^ cells in dorsal horns of the same groups. (D) Quantification of Iba^+^/pp38^+^ cells in dorsal horns of the same groups. Values are the mean ± SEM of 4-5 mice per group. * p<0.05, **p<0.01, same group, day comparisons, two-way ANOVA with Tukey multiple-comparison test*.*


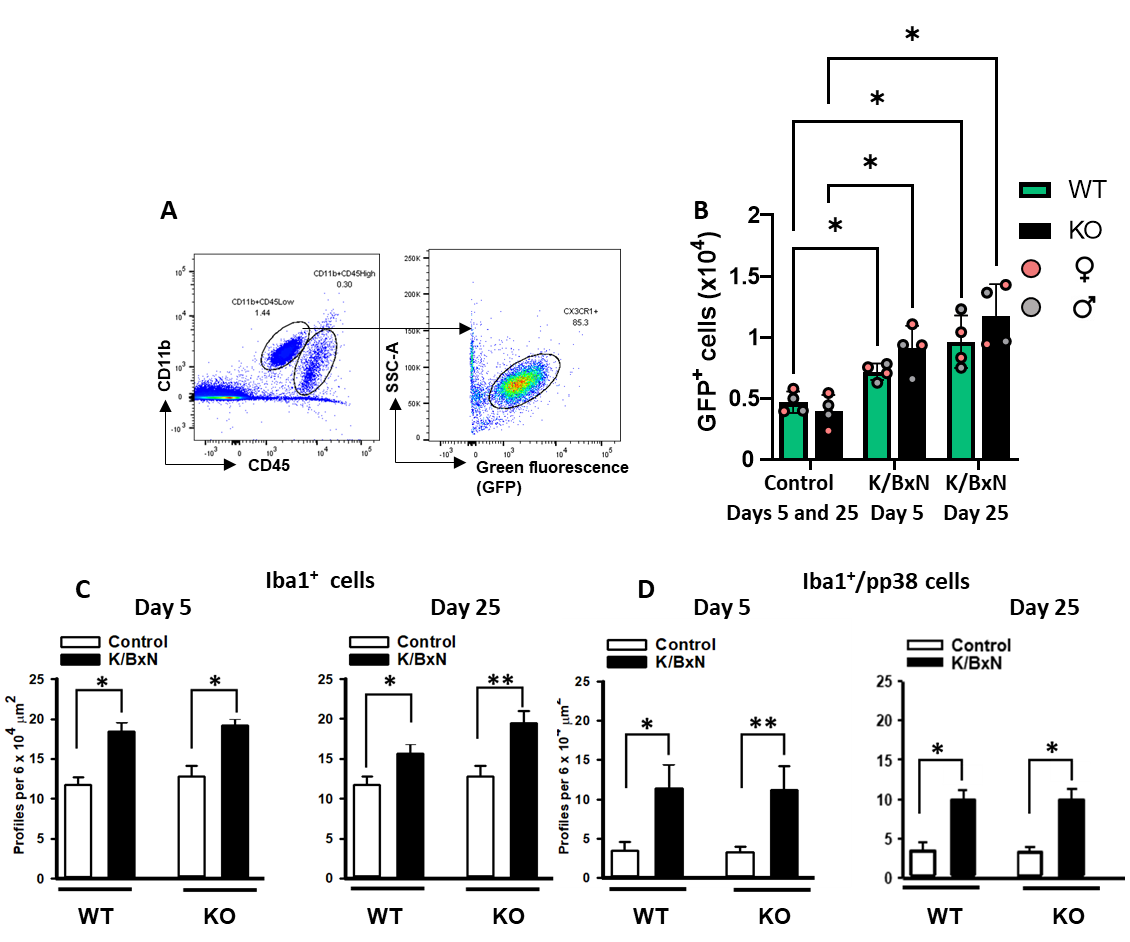


**Supplementary Figure 3. K/BxN serum transfer arthritis is associated with infiltration of monocytes and macrophages in hind paws which is not altered in CX_3_CR_1_ KO.**  (A) Dynamics of individual cell subsets in the hind paws during arthritis induced by K/BxN serum transfer. (B-C) M2/M1 and M1/M2 ratios of macrophages in hind paws of WT and KO at day 5 and day 25 after K/BxN serum transfer. Values are the mean ± S.D. of 8 mice per group. * p<0.05, **p<0.01, same group, day comparisons, one-way ANOVA, post-hoc Bonferroni.


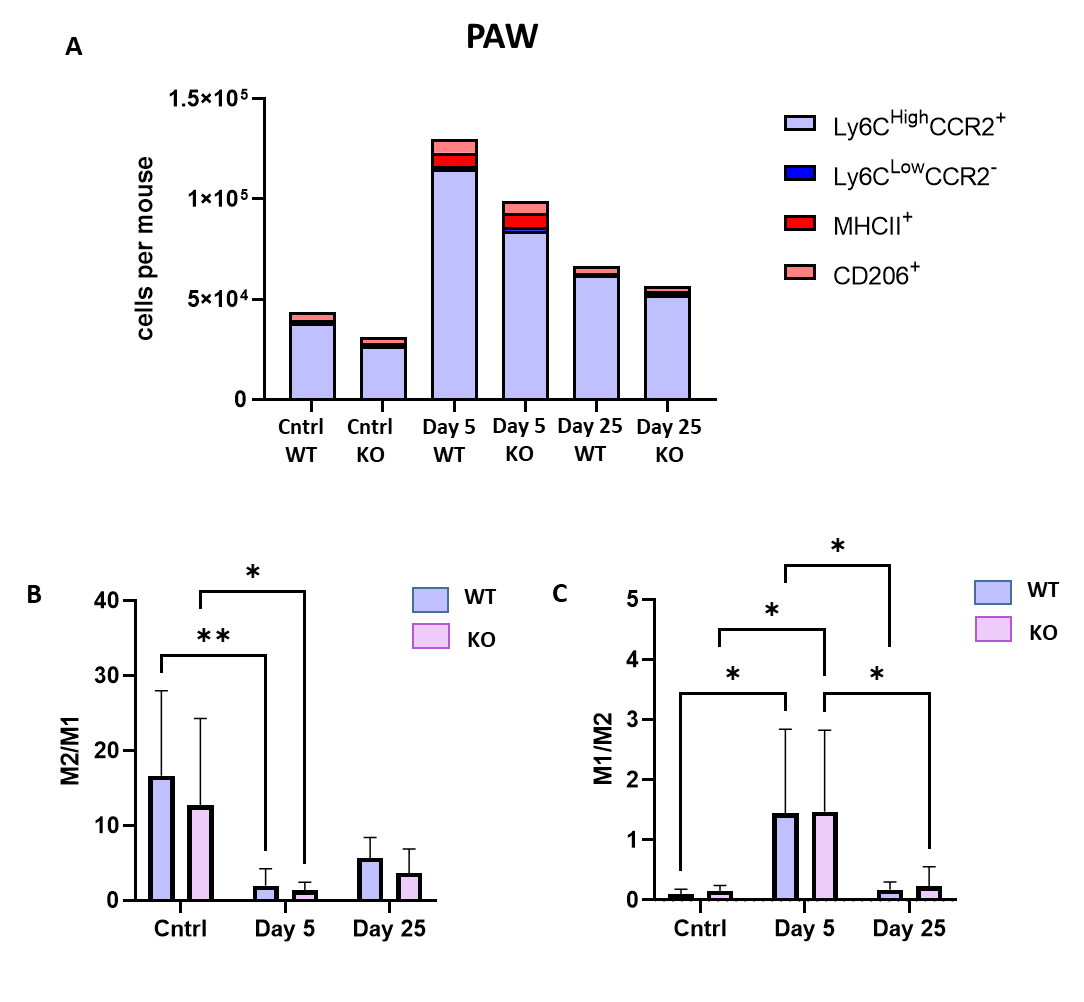


**Supplementary Figure 4. K/BxN serum transfer arthritis is associated with infiltration of monocytes and macrophages in WT, but not KO DRG** (A) Dynamics of monocyte subsets in DRG at day 5 and day 25 after K/BxN serum transfer. (B) Dynamics of macrophage subsets in DRG after K/BxN serum transfer. (C-D) M2/M1 and M1/M2 ratios of macrophages in WT and KO DRG. Values are the mean ± S.D. of 8 mice per group. * p<0.05, **p<0.01, same group, day comparisons, one-way ANOVA, post-hoc Bonferroni.


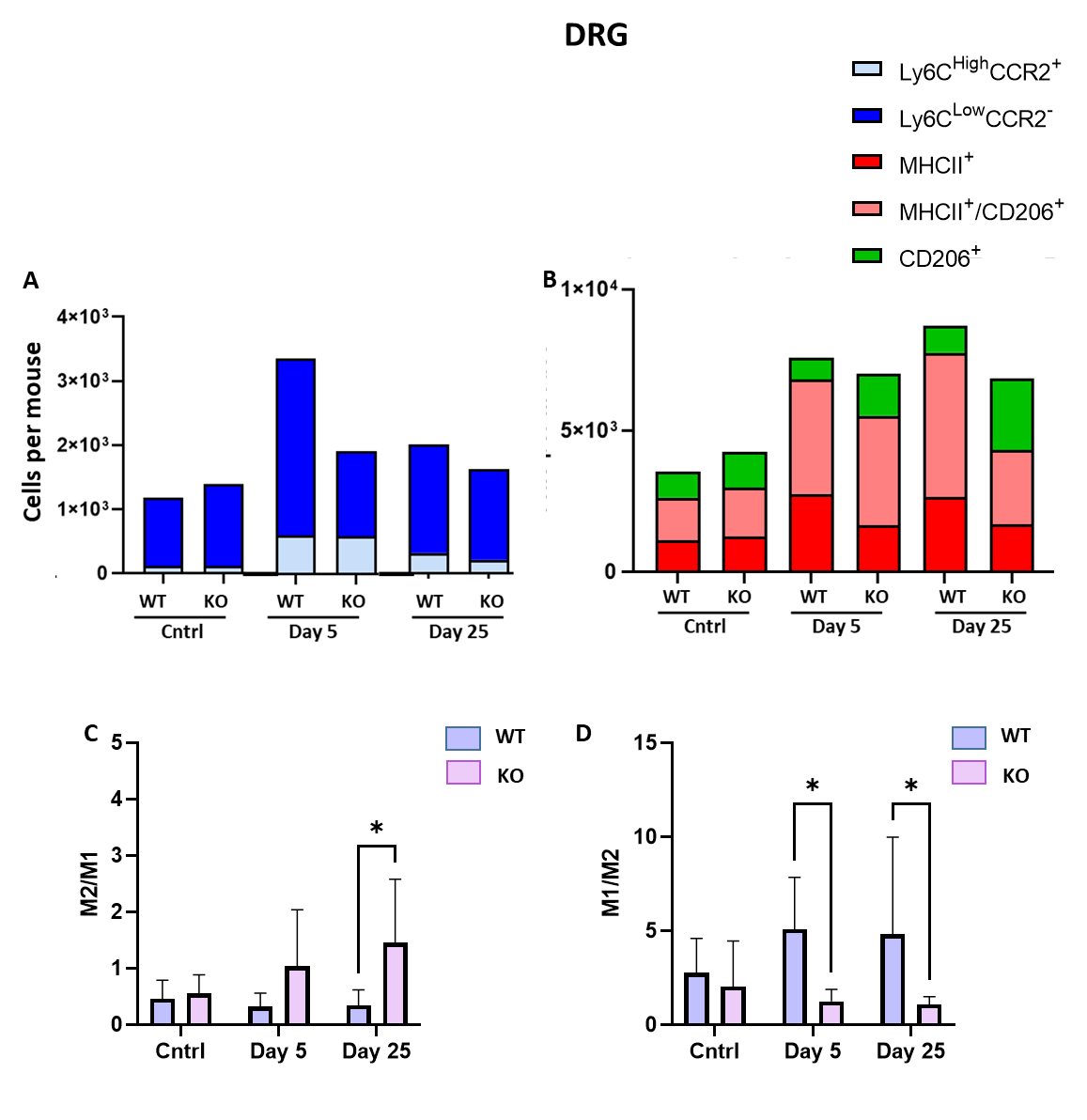


**Supplementary Figure 5.FKN expression is reduced in HUVEC upon stimulation with CRGP.** HUVEC treated with Vehicle, CGRP (1μM), or CGRP^+^ CGRP 8-37(100 μM) were stained with CD31 (red), FKN (green) and DAPI (blue). (A) Representative images of HUVEC displaying CD31, FKN and DAPI fluorescence when treated with Vehicle, CGRP, or CGRP^+^ CGRP 8-37 respectively. (B) Representative figure of Fluorescence Minus One control (FMO) of FKN staining. (C) Representative histogram plot of FKN MFI in HUVEC cells incubated with CGRP (light blue) or vehicle (red). (D-G) Bar charts present MFI units for FKN, ICAM, VCAM and e-selectin (CD62-e) of HUVEC. Data are means ± S.D., n = 5 for each group. *p< 0.05, **p < 0.01, ***p<0.001 one-way ANOVA, post hoc Bonferroni.


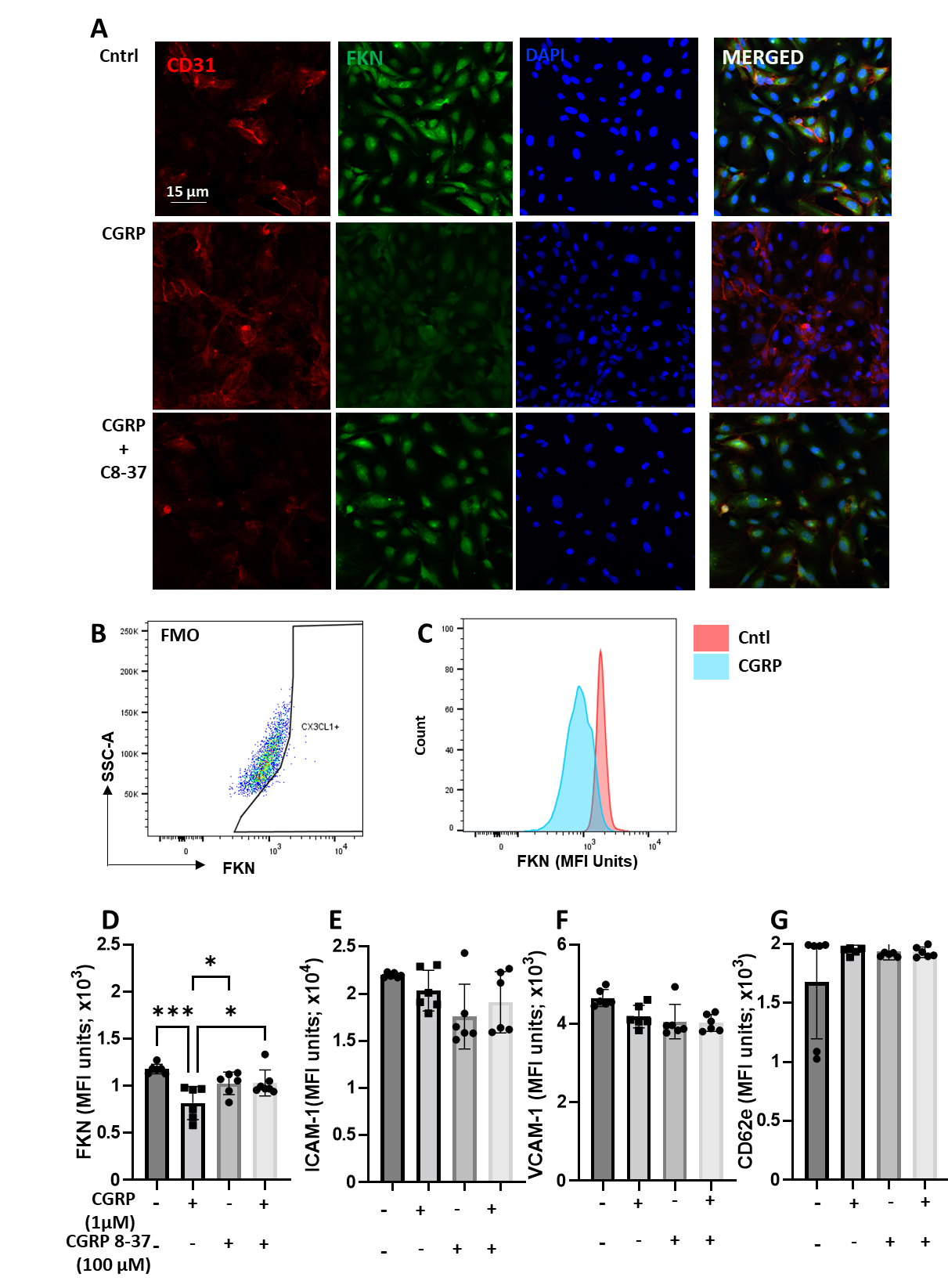


**Supplementary Figure 6. Schematic representation of mechanism of DRG monocytes/macrophages regulating arthritis pain.** Inflammatory arthritis induces the expression of selected adhesion molecules, which promote monocyte trafficking. (1) CX_3_CR_1_ monocyte binds transmembrane FKN. (2) CGRP released by neurons induces expression of endothelial ADAM-17 which liberates soluble FKN chemokine domain (3). sFKN activation of CX_3_CR_1_ receptors in monocytes induces infiltration (4), and activation of macrophages that release pro-nociceptive cytokine IL-6 (5), which in turn sensitises neurons via activation of gp-130 (6).


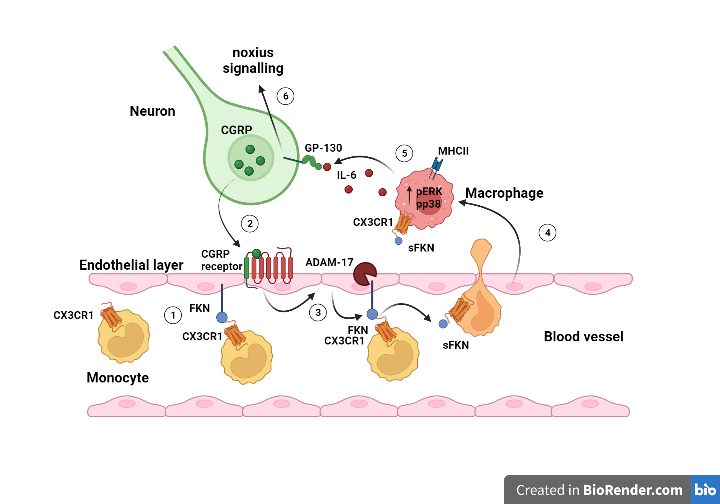

Supplement: Supplementary data 1 [file mmc1.docx]
